# Three-Dimensional Finite Element Analysis of Anterior Single Implant-Supported Prostheses with Different Bone Anchorages

**DOI:** 10.1155/2015/321528

**Published:** 2015-08-13

**Authors:** Fellippo Ramos Verri, Joel Ferreira Santiago Júnior, Daniel Augusto de Faria Almeida, Ana Caroline Gonçales Verri, Victor Eduardo de Souza Batista, Cleidiel Aparecido Araujo Lemos, Pedro Yoshito Noritomi, Eduardo Piza Pellizzer

**Affiliations:** ^1^Department of Dental Materials and Prosthodontics, Universidade Estadual Paulista (UNESP), 1193 Jose Bonifacio Street, 16015-050 Araçatuba, SP, Brazil; ^2^Department of Health Sciences, University of Sacred Heart (USC), Bauru, SP, Brazil; ^3^Department of Pediatric and Community Dentistry, Dental School of Araçatuba, Universidade Estadual Paulista (UNESP), 15015-050 Araçatuba, SP, Brazil; ^4^Renato Archer Research Center (CTI), Campinas, SP, Brazil

## Abstract

The aim of this study was to evaluate the stress distribution of monocortical and bicortical implant placement of external hexagon connection in the anterior region of the maxilla by 3D finite element analysis (FEA). 3D models were simulated to represent a bone block of anterior region of the maxilla containing an implant (4.0 × 10.0 mm) and an implant-supported cemented metalloceramic crown of the central incisor. Different techniques were tested (monocortical, bicortical, and bicortical associated with nasal floor elevation). FEA was performed in FEMAP/NeiNastran software using loads of 178 N at 0°, 30°, and 60° in relation to implant long axis. The von Mises, maximum principal stress, and displacement maps were plotted for evaluation. Similar stress patterns were observed for all models. Oblique loads increased the stress concentration on fixation screws and in the cervical area of the implants and bone around them. Bicortical technique showed less movement tendency in the implant and its components. Cortical bone of apical region showed increase of stress concentration for bicortical techniques. Within the limitations of this study, oblique loading increased the stress concentrations for all techniques. Moreover, bicortical techniques showed the best biomechanical behavior compared with monocortical technique in the anterior maxillary area.

## 1. Introduction

Adequate primary stability of osseointegrated implants is considered one of the most important rules for the success of rehabilitation using dental implants [1, 2]. Among factors that influence the primary stability, the bicorticalization of the implant has been considered by some studies [2, 3]. Initially, bicorticalization was used to improve the stability of implant on the bone tissue for anterior and in the posterior region of the maxilla [3], mainly for immediate loading procedures [2]. In the low-density bone (worst prognosis for osseointegration), this technique could be effective when it is associated with underpreparation of implant socket, resulting in the increase of primary stability [4]. However, there is no consensus if this increase of primary stability could reduce the stress distribution on bone tissue.

Researches indicate higher stress and strain concentrations in low-quality bone tissue [5, 6]. In this bone type, in upper jaw, bicorticalization placement of the implant could allow the use of a longer implant, improving the stress and strain distribution. Some authors believe in its beneficial improvement for implantology [2, 4]. On the contrary, some studies indicate probable bone atrophy by disuse on medium region of the implant due to increase of stress concentration on occlusal and apical cortical bones and decrease of stress in the medium implant region [7–9]. Hence, advantage of bicorticalization technique is not a consensus yet.

Retrospective clinical study indicates lower survival rate of bicorticalized implants compared to monocortical implants [7]. On the other hand, clinical studies showed high predictability of implants placed at nasal floor, reaching survival rate of 96% in two to five years of follow-up [10]. Recently, cohort retrospective study of 32 patients that received 100 implants placed at anterior region of the maxilla associated with nasal floor elevation indicated survival rate of 100% in 27 months of follow-up. It was pointed out that this high survival rate occurred due to bicortical stabilization on nasal floor [11]. However, few studies evaluated biomechanical behavior of bicorticalization in the anterior region of the maxilla. In a biomechanical point of view, the stress distribution around bicorticalized implants is extremely relevant to indicating the best surgical technique for implant placement, mainly to predict overload tendency of implant-supported prostheses fabricated after implant placement.

Computerized simulation has been widely used for biomechanical evaluation in implantology [12–16], including some analyses of anterior region of the maxilla [17, 18]. Finite element analysis (FEA) is one of the most used techniques. Thus, the aim of this study was to compare the biomechanical behaviors of single implant-supported prostheses in the anterior region of the maxilla comparing surgical techniques of implant placement.

## 2. Materials and Methods

### 2.1. Three-Dimensional Modeling

**Figure 1:**
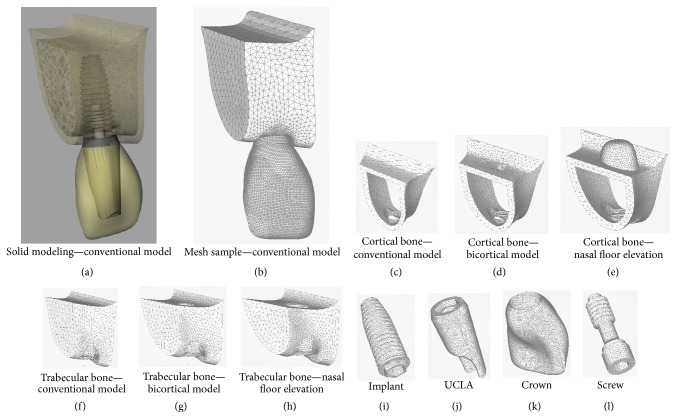
Solid modeling and generated mesh samples of all structures. (a) Modeling of model 1; (b) meshes of model 1; (c) mesh of cortical bone of model 1; (d) mesh of cortical bone of model 2; (e) mesh of cortical bone of model 3; (f) mesh of trabecular bone of model 1; (g) mesh of trabecular bone of model 2; (h) mesh of trabecular bone of model 3; (i) mesh of implant of all models; (j) mesh of UCLA of all models; (k) mesh of crown of all models; (l) mesh of screw of all models.

The modeling methodology was based on previous studies [12–15]. For this study three 3D models were created (Table 1, Figure 1). Each model presented a bone block of upper right central incisor area where an implant was positioned to support a single cemented crown. The implant simulated was an external hexagon implant type with dimensions of 4.0 × 10.0 mm (Conexão Sistemas de Prótese Ltda, Arujá, Brazil).

The geometry of bone tissue was obtained by CT-Scan recomposition of transversal images of anterior region of the maxilla by aid of InVesalius 3.0 software (CTI, São Paulo, Brazil). The finishing and simplifying of surfaces were made in Rhinoceros 4.0 software (NURBS modeling for Windows, Robert McNeel & Associates, Seattle, USA), including bone division of cortical bone layer of 1 mm surrounding trabecular bone. The bone density was considered as type III bone [19]. Model 1 (monocortical anchorage) presented bone height of 12 mm and remaining bone of 2 mm height over the implant apex (Figures 1(c) and 1(f)). Model 2 (bicortical anchorage) presented bone height of 10 mm with the implant apex placed at apical cortical bone (nasal floor) (Figures 1(d) and 1(g)). Model 3 (bicortical anchorage associated with nasal floor elevation) presented 8 mm of bone height remaining 2 mm of cortical bone surrounding the apex that was placed at apical area of nasal floor similarly as described by Summers for sinus lift technique (Figures 1(e) and 1(h)) [20].

Geometries of implant and its components (UCLA and screw) were obtained by simplification of its original design of external hexagon type (Conexão Sistemas de Prótese Ltda., Arujá, Brazil) in the SolidWorks 2010 (SolidWorks Corp., Massachusetts, USA) and Rhinoceros 4.0 software (Figures 1(i), 1(j), and 1(l)).

Metal-ceramic crown was constructed from superficial scanning of occlusal surface of an artificial tooth (upper central incisor) obtained from a dental mannequin with the assistance of a surface scanner (3D MDX-20, Roland DG, Shizuoka-ken, Japan) according to previous studies [15]. Then, in Rhinoceros 4.0 real dimensions of the crown were established simulating an average thickness of 1 mm of feldspathic ceramic involving the infrastructure that was joined to internal surface of UCLA modeled as cited previously (Figure 1(k)). The modeled crown was positioned together with the implant and screw in the bone block.

### 2.2. Finite Element Analysis Configuration

All geometries were exported to FEMAP 11.0 software (Siemens PLM Software Inc., California, USA) for finite element preprocessing phase. The meshes were obtained using parabolic solid elements (Figures 1(b)–1(l)). Mechanical properties of all simulated materials were attributed to the generated meshes according to literature data (Table 2) and all materials were considered isotropic, homogeneous, and linearly elastic.

Symmetric welds were simulated for all contacts, with the exception of the abutment/implant contact, which was simulated by symmetric contact. Restrictions were assumed as fixed in *x*, *y*, and *z* direction and applied at construction lines of the upper region of the bone block in the nasal floor region for each model. The applied force was 178 N, at 0°, 30°, and 60°, in relation to implant long axis at palatine surface of the incisor according to reference study [18].

NeiNastran 11 software (Noran Engineering, Inc., California, USA) performed the finite element solutions. All solutions were exported to FEMAP 11.0 for postprocessing and visualization of stress maps and displacement values in areas of interest for analysis. The solid mesh convergence error values were obtained for all structures of interest to the study and can be seen in Table 3.

### 2.3. Criteria of Stress Analysis

Displacement patterns were used to verify the tendency of movement of all models. Some values (in millimeters) of specific areas were obtained for comparison between models. Maximum principal stress was used to analyze stress on bone tissue as recommended to analyze compression and traction patterns of friable materials [12–16]. The von Mises stress was used to analyze implants and its components as recommended to analyze ductile solid materials [14, 15]. Both analyses have units in Mega Pascal (MPa).

## 3. Results

### 3.1. von Mises Stress Analysis

**Figure 2:**
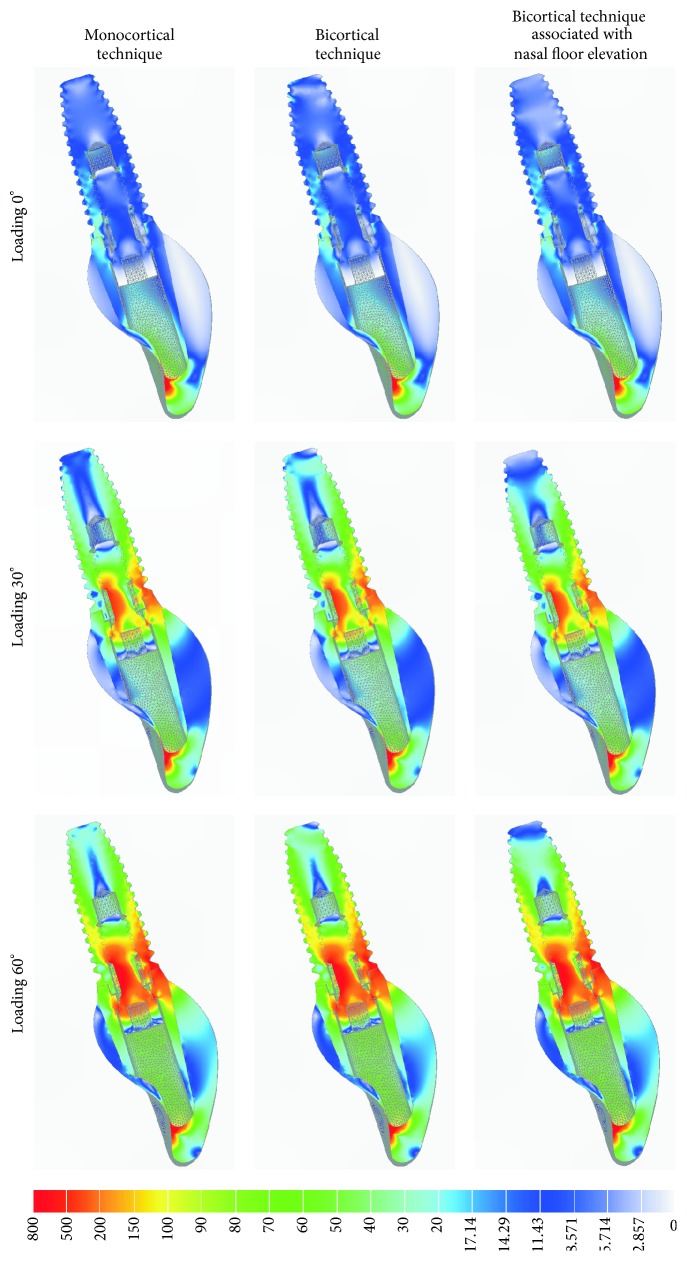
The von Mises stress analysis of implant, UCLA, crown, and screw fixation in sagittal view for different techniques.

The von Mises criteria showed stress concentration near UCLA/implant interface and in the fixation screw. The pattern of distribution was similar for all models. However, the increase of loading direction (0° to 60°) generated higher stress concentrations. No differences of von Mises stress were observed among models in the cervical region of the implant/crown. Apical region of the implant of bicortical technique associated with nasal floor elevation presented less concentration of stress (Figure 2).

### 3.2. Maximum Principal Stress Analysis

**Figure 3:**
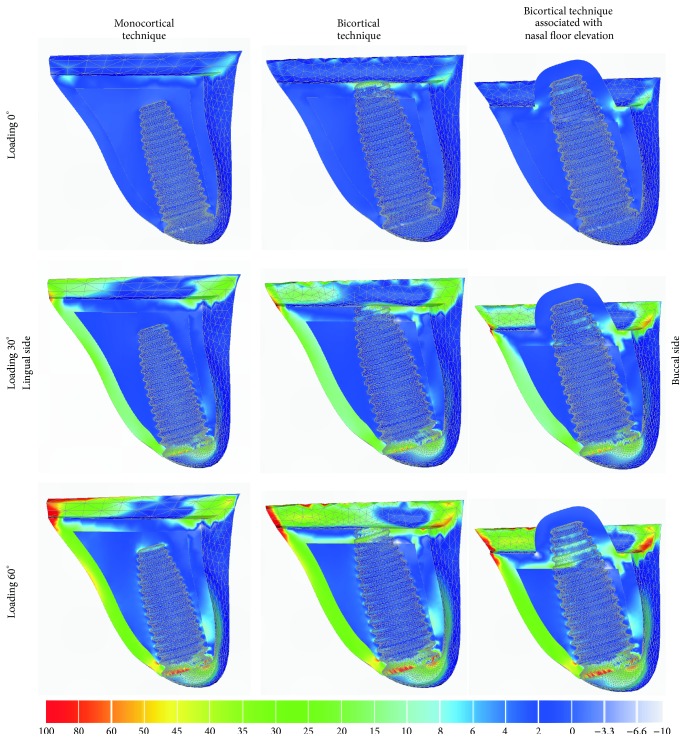
Maximum principal stress analysis of bone tissue in sagittal view for different techniques.

**Figure 4:**
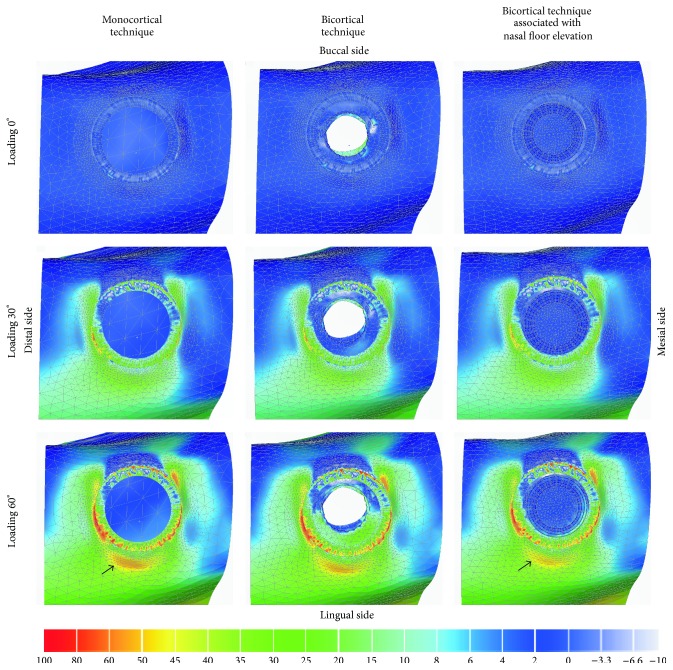
Maximum principal stress analysis of cortical bone in occlusal view for different techniques.

In general, stress of compression was located at buccal side of the bone in contact with the cervical collar of the implant and stress of traction was located in the opposite side (Figures 3 and 4). Sagittal view (Figure 3) showed similar patterns of stress distribution for all techniques, mainly near to implant neck area. The bicortical techniques showed higher stress concentration of traction on bone in the implant apex area as compared to that of monocortical technique. The increase of the loading inclination (0° to 60°) showed higher stress concentration, reaching over 100 MPa in some areas including the area surrounding the collar of the implant.

By occlusal view (Figure 4), the models showed higher stress concentration surrounding the implant neck, mainly for 60° of loading direction. Moreover, bicortical technique associated with nasal floor elevation showed less extensive area of stress of traction at lingual side as compared to that of monocortical anchorage technique (Figure 4, arrows, orange area).

### 3.3. Displacement Analysis

**Figure 5:**
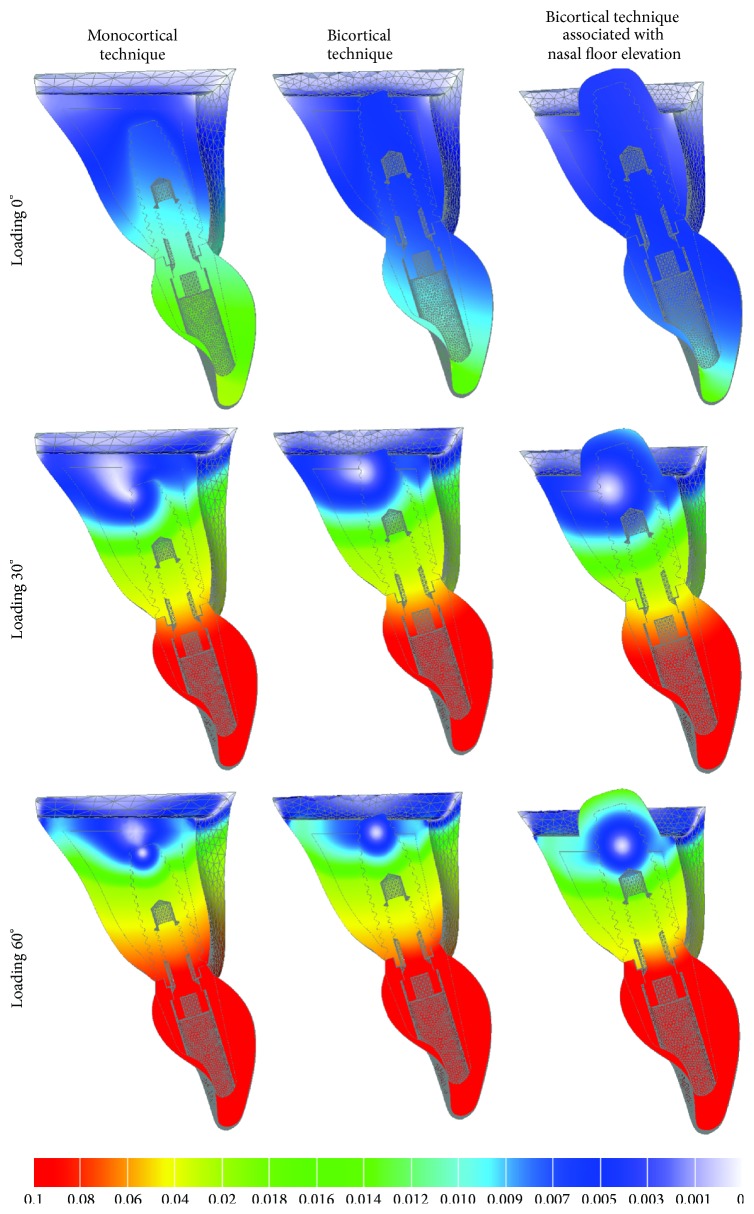
Displacement analysis of all models in sagittal view for different techniques.

All models presented similar pattern of displacement distribution (Figure 5). The increase of the loading inclination (0° to 60°) tends to increase the displacement tendency. Bicortical technique associated with nasal floor elevation showed smallest values for all analyzed regions followed by bicortical technique and conventional technique (Table 4). In some regions, as cortical bone, the reduction of displacement tendency was 50% for bicortical technique association with nasal floor elevation as compared to that for conventional technique.

## 4. Discussion

In this study, bicorticalization techniques seemed more effective biomechanically because they reduced the stress distribution in the bone tissue in the area around collar of the implant and showed less tendency of displacement. Literature has emphasized the importance of primary stability for osseointegration, mainly due to favoring the osteogenesis and bone turnover [2]. Therefore, adequate primary stability is an indispensable condition for surgical procedures of implant placement [2], principally in clinical situation of immediate loading of implants [21]. Consider that bicorticalization increases significantly the removal torque as compared to that of monocortical anchorage of implants [2]; consequently, there is an improvement of biomechanical stability using bicortical technique [22].

Bicorticalization techniques offered possibility of transmission of stress to upper cortical bone (in the nasal floor) dissipating stress transferred by occlusal loading. Thus, bicorticalized bone tissue could act as biologic mechanism to prevent occlusal overload [23]. Similar methodology indicated higher stress reduction of bicortical technique as compared to that of monocortical anchorage technique [24]. This information agrees with Huang et al., 2009 [25], who demonstrated that a bicorticalized implant of ≥8.5 mm-long decreased the stresses in both cortical and trabecular bone by 50% compared to a monocortical implant. Thereby, benefits of bicorticalization are expected such as reduction of stress levels in low quality bone as found in the anterior region of the maxilla and this occurred in this study.

Studies showed that oblique forces are more dangerous to the peri-implant bone tissue [12–15, 18, 24], especially for rehabilitation using external hexagon implants [12]. In our results, 60° of loading direction showed higher stress concentration. External hexagon implants concentrate stress nearest implant neck region as compared to other internal connections [12] and this tendency was observed in our study. Once again this fact could explain higher tendency for screw fracture and/or loosening in external hexagon implants [12]. Considering this type of implant and conditions of the study, the results indicate that bicorticalized implants have similar stress distribution compared to monocortical placement. However, the movement tendency is lower for bicortical techniques in this area and this fact could contribute to decreasing the loss of tightening of fixation screws. Other connections could show different results; thus, further studies should be performed to establish this comparison. Moreover, compressive and tractive stress also presented similar results to the literature [18].

Despite disuse atrophy cited in adjacent areas of cortical bones locked by an implant [7, 9] our results showed higher levels of stress concentrations localized at upper cortical bone with no significant differences in stress patterns of trabecular bone, showing only slight reduction in tractive stress when considering bicortical technique in the lingual region of cortical bone. Thus, considering finite element analysis as a static analysis, it is not possible to determine biological evidences, as well as correlation with disuse atrophy, as suggested by some studies [7–9].

In this study, the apical bone tissue involving the implant was simulated as mature cortical bone and could have contributed to the results. There is no published study clearing the density of grafted bone after this technique. Findings of biomechanical simulation suggest that the graft quality changes the biomechanical performance of implants and it is critical to consider the clinical situations where poor grafted bone quality has been observed [26]. In this way, as this simulation considered optimal performance of the bone graft, these results should be analyzed with care since several troubles are reported regarding this technique as bleeding, swelling, pain, hematoma, graft infection, implant displacement, rhinitis, and sinusitis [11, 27, 28]. Certainly, any of these situations mentioned will not result in a good bone graft.

Although citations of bicorticalized implants have less survival rate by some studies [7] and good predictability by others [10], even increasing the survival rate [11], no analysis of bruxism, antagonist arch, cantilever extension, parafunctions, or other factors was investigated. Therefore, results of these studies could be carried out carefully. In this study it was indicated that bicorticalized implants are mechanically viable as rehabilitation option.

FEA studies possess limitations since it is a computational simulation and factors as restrictions of models, materials properties, load values, and application type could change the results and are limited as compared to clinical evaluations. However, this technique allows adequate biomechanical comparative study of regions of bone/implant interface in different situations, suggesting the more suitable situation under a biomechanical point of view. Controlled randomized clinical trials should be performed to evaluate and compare the described technique.

## 5. Conclusions

Considering the limitations of this study

1. oblique loading increased the stress concentrations independently of the simulated surgical technique of implant placement;
2. qualitative analysis of stress and displacement showed that bicortical techniques showed the best biomechanical behavior compared with monocortical technique in the anterior maxillary area.

## Figures and Tables

**Table 1:** Description of models developed for this study.

Implant	Surgical technique	Loading
4.0 × 10.0 mm external hexagon	Model 1	0°
	Monocortical anchorage	30°
	Conventional placement	60°
	Model 2	0°
	Bicortical bone anchorage	30°
	Bicortical placement	60°
	Model 3	0°
	Bicortical bone anchorage	30°
	Bicortical placement associated with nasal floor elevation	60°

**Table 2:** Mechanical properties of all simulated materials [14, 15].

Material	Elastic modulus (*E* – Gpa)	Poisson's ratio (*v*)
Cortical bone	13.7	0.3
Trabecular bone	1.37	0.3
Titanium	110	0.35
NiCr alloy	206	0.33
Feldspathic porcelain	82.8	0.35

**Table 3:** Solid mesh convergence error.

Models	Loading	Implant	Screw	Cortical bone	Trabecular bone
Conventional technique	0°	0,00026	0,00011	0,00024	0,00008
	30°	0,00390	0,00442	0,00159	0,00014
	60°	0,00870	0,00970	0,00280	0,00018

Bicortical technique	0°	0,00033	0,00011	0,00043	0,00004
	30°	0,00409	0,00465	0,00209	0,00012
	60°	0,00947	0,01032	0,00381	0,00018

Nasal floor elevation	0°	0,00030	0,00012	0,00045	0,00006
	30°	0,00416	0,00477	0,00218	0,00014
	60°	0,00944	0,01056	0,00333	0,00019

**Table 4:** Description values of models (in millimeter).

Surgical technique	Loading	Implant	Screw	Cortical bone	Trabecular bone
Conventional technique	0°	0,012	0,013	0,012	0,012
	30°	0,057	0,101	0,056	0,045
	60°	0,101	0,194	0,099	0,079

Bicortical technique	0°	0,008	0,009	0,008	0,007
	30°	0,046	0,089	0,046	0,035
	60°	0,081	0,172	0,081	0,062

Nasal floor elevation	0°	0,006	0,007	0,006	0,006
	30°	0,037	0,077	0,036	0,026
	60°	0,065	0,150	0,064	0,045

## References

[1] Degidi M., Daprile G., Piattelli A. (2010). Determination of primary stability: a comparison of the surgeon's perception and objective measurements. *The International Journal of Oral & Maxillofacial Implants*.

[2] Ahn S.-J., Leesungbok R., Lee S.-W., Heo Y.-K., Kang K. L. (2012). Differences in implant stability associated with various methods of preparation of the implant bed: an *in vitro* study. *Journal of Prosthetic Dentistry*.

[3] Ferrigno N., Laureti M., Fanali S. (2006). Dental implants placement in conjunction with osteotome sinus floor elevation: a 12-year life-table analysis from a prospective study on 588 ITI implants. *Clinical Oral Implants Research*.

[4] Martinez H., Davarpanah M., Missika P., Celletti R., Lazzara R. (2001). Optimal implant stabilization in low density bone. *Clinical Oral Implants Research*.

[5] Tada S., Stegaroiu R., Kitamura E., Miyakawa O., Kusakari H. (2003). Influence of implant design and bone quality on stress/strain distribution in bone around implants: a 3-dimensional finite element analysis. *International Journal of Oral and Maxillofacial Implants*.

[6] Lin C.-L., Wang J.-C., Ramp L. C., Liu P.-R. (2008). Biomechanical response of implant systems placed in the maxillary posterior region under various conditions of angulation, bone density, and loading. *International Journal of Oral and Maxillofacial Implants*.

[7] Ivanoff C.-J., Gröndahl K., Bergström C., Lekholm U., Brånemark P.-I. (2000). Influence of bicortical or monocortical anchorage on maxillary implant stability: a 15-year retrospective study of Branemark System implants. *International Journal of Oral and Maxillofacial Implants*.

[8] Clelland N. L., Lee J. K., Bimbenet O. C., Gilat A. (1993). Use of an axisymmetric finite element method to compare maxillary bone variables for a loaded implant.. *Journal of Prosthodontics*.

[9] Rieger M. R., Mayberry M., Brose M. O. (1990). Finite element analysis of six endosseous implants. *Journal of Prosthetic Dentistry*.

[10] Brånemark P. I., Adell R., Albrektsson T., Lekholm U., Lindström J., Rockler B. (1984). An experimental and clinical study of osseointegrated implants penetrating the nasal cavity and maxillary sinus. *Journal of Oral and Maxillofacial Surgery*.

[11] Mazor Z., Lorean A., Mijiritsky E., Levin L. (2012). Nasal floor elevation combined with dental implant placement. *Clinical Implant Dentistry and Related Research*.

[12] de Faria Almeida D. A., Pellizzer E. P., Verri F. R., Santiago J. F., de Carvalho P. S. P. (2014). Influence of tapered and external hexagon connections on bone stresses around tilted dental implants: three-dimensional finite element method with statistical analysis. *Journal of Periodontology*.

[13] Santiago Junior J. F., Pellizzer E. P., Verri F. R., de Carvalho P. S. P. (2013). Stress analysis in bone tissue around single implants with different diameters and veneering materials: a 3-D finite element study. *Materials Science and Engineering C*.

[14] Ramos Verri F., Santiago Junior J. F., de Faria Almeida D. A. (2015). Biomechanical influence of crown-to-implant ratio on stress distribution over internal hexagon short implant: 3-D finite element analysis with statistical test. *Journal of Biomechanics*.

[15] Verri F. R., Batista V. E. D. S., Santiago J. F., Almeida D. A. D. F., Pellizzer E. P. (2014). Effect of crown-to-implant ratio on peri-implant stress: a finite element analysis. *Materials Science & Engineering C: Materials for Biological Applications*.

[16] Akay C., Yalug S. (2015). Biomechanical 3-dimensional finite element analysis of obturator protheses retained with zygomatic and dental implants in maxillary defects. *Medical Science Monitor*.

[17] Caglar A., Bal B. T., Aydin C., Yilmaz H., Ozkan S. (2010). Evaluation of stresses occurring on three different zirconia dental implants: three-dimensional finite element analysis. *The International Journal of Oral & Maxillofacial Implants*.

[18] Hsu M.-L., Chen F.-C., Kao H.-C., Cheng C.-K. (2007). Influence of off-axis loading of an anterior maxillary implant: a 3-dimensional finite element analysis. *International Journal of Oral and Maxillofacial Implants*.

[19] Lekholm U., Zarb G. (1985). *Patient Selection and Preparation*.

[20] Summers R. B. (1994). The osteotome technique: part 3—less invasive methods of elevating the sinus floor. *Compendium*.

[21] Strub J. R., Jurdzik B. A., Tuna T. (2012). Prognosis of immediately loaded implants and their restorations: a systematic literature review. *Journal of Oral Rehabilitation*.

[22] Xiao J. R., Li Y. Q., Guan S. M. (2012). Effects of lateral cortical anchorage on the primary stability of implants subjected to controlled loads: an in vitro study. *British Journal of Oral and Maxillofacial Surgery A (Scotland)*.

[23] Van Oosterwyck H., Duyck J., Sloten J. V. (1998). The influence of bone mechanical properties and implant fixation upon bone loading around oral implants. *Clinical Oral Implants Research*.

[24] Chang S.-H., Lin C.-L., Lin Y.-S., Hsue S.-S., Huang S.-R. (2012). Biomechanical comparison of a single short and wide implant with monocortical or bicortical engagement in the atrophic posterior maxilla and a long implant in the augmented sinus. *The International Journal of Oral & Maxillofacial Implants*.

[25] Huang H.-L., Fuh L.-J., Ko C.-C., Hsu J.-T., Chen C.-C. (2009). Biomechanical effects of a maxillary implant in the augmented sinus: a three-dimensional finite element analysis. *The International Journal of Oral & Maxillofacial Implants*.

[26] Inglam S., Suebnukarn S., Tharanon W., Apatananon T., Sitthiseripratip K. (2010). Influence of graft quality and marginal bone loss on implants placed in maxillary grafted sinus: a finite element study. *Medical and Biological Engineering and Computing*.

[27] Felisati G., Chiapasco M., Lozza P. (2013). Sinonasal complications resulting from dental treatment: outcome-oriented proposal of classification and surgical protocol. *The American Journal of Rhinology & Allergy*.

[28] Kfir E., Kfir V., Goldstein M., Mazor Z., Kaluski E. (2012). Minimally invasive subnasal elevation and antral membrane balloon elevation along with bone augmentation and implants placement. *Journal of Oral Implantology*.

